# Supplementary material for: Toward stabilization of formamidinium lead iodide perovskites by defect control and composition engineering
Source: Nat Commun. 2024 Feb 24;15:1707. doi: 10.1038/s41467-024-46044-x (PMC10894298; doi:10.1038/s41467-024-46044-x)
Supplement: Supplementary file 1 — Supplementary information [file 41467_2024_46044_MOESM1_ESM.pdf]

Supplementary Information

Toward stabilization of formamidinium lead iodide perovskites by defect control and composition engineering

Yuhang Liang,[1,2,*] *Feng Li,*[2,*], *Xiangyuan Cui,*[3,*] *Taoyuze Lv,*[2] *Catherine Stampfl,*[2] *Simon P. Ringer,*[3] *Xudong Yang,*[4,5,6] *Jun Huang,*[1,*] *Rongkun Zheng*[2,*]

Yuhang Liang,[1,2] *Feng Li,*[2,*], *Xiangyuan Cui,*[3,*] *Taoyuze Lv,*[2] *Catherine Stampfl,*[2] *Simon P. Ringer,*[3] *Xudong Yang,*[4,5,6] *Jun Huang,*[1,*] *Rongkun Zheng*[2,*]

[1] School of Chemical and Biomolecular Engineering, The University of Sydney, NSW 2006, Australia

[2] School of Physics, The University of Sydney, NSW 2006, Australia

[3] School of Aerospace, Mechanical and Mechatronic Engineering, The University of Sydney, NSW 2006, Australia

[4] State Key Laboratory of Metal Matrix Composites, Shanghai Jiao Tong University, Shanghai 200240, China

[5] Center of Hydrogen Science, School of Materials Science and Engineering, Shanghai Jiao Tong University, Shanghai 200240, China

[6] Zhangjiang Institute for Advanced Study, Shanghai Jiao Tong University, Shanghai 201210, China

*Yuhang Liang: yuhang.liang@sydney.edu.au

*Feng Li: feng.li2@sydney.edu.au

*Xiangyuan Cui: carl.cui@sydney.edu.au

*Jun Huang: jun.huang@sydney.edu.au

*Rongkun Zheng: rongkun.zheng@sydney.edu.au

Supplementary Notes

The details for SCLC analyses

We also performed current–voltage (*I*–*V*) measurements to assess the charge transport properties and trap behaviors of both Cs- and Cs-Eu mixed-cation-doped FA-based perovskite single crystals. The space-charge-limited-current (SCLC) analyses were carried out based on the obtained *I*-*V* data. As shown in **Figures 6f-i** in the main text, we can clearly find that three regions were identified in the *I*–*V* curves: Ohmic (n = 1), trap-filling (n > 3), and Child's (n = 2) regions.

At the low applied voltage, an Ohmic region is confirmed. Following the first region, it exhibits a rapid nonlinear rise in current starting at the point of trap-filled limit (TFL) voltage V_{TFL}, indicating the transition into the trap-filling region. In the second region, all the trap states are expected to be filled by the injected carriers. The trap density (N_t) can be evaluated by the following equation:

$$N_t = \frac{2V_{TFL}\varepsilon\varepsilon_0}{ed^2}$$

in which ε is the relative dielectric constant materials, ε_0 is the vacuum permittivity, *e* is the electronic charge, and *d* represents the thickness of the single crystals.

The carrier mobility (μ) could be extracted from the third region (Child's region) that shows trap-free characteristic at high bias, following the Mott–Gurney's SCLC theory expressed by the following equation:

$$\mu = \frac{8d^3}{9\varepsilon\varepsilon_0}\frac{\partial J}{\partial(V^2)}$$

where *J* is current density and *V* is the corresponding applied voltage.

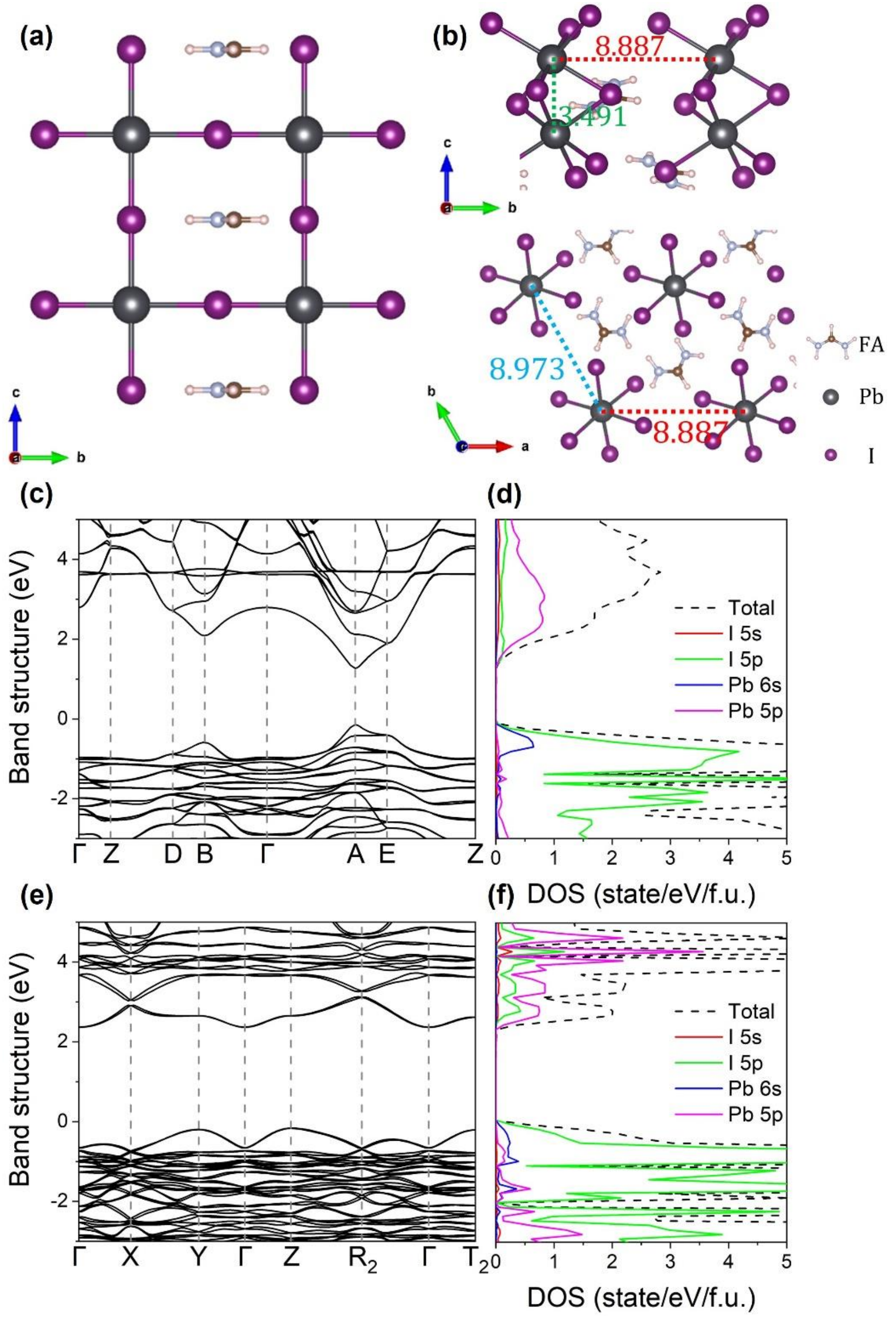

Supplementary Figure 1. Optimized structures and electronic structures. Optimized structures of the (**a**) α-phase and (**b**) δ-phase of $FAPbI_3$. (**c**) Calculated band structure and (**d**) the density of state (DOS) of α-$FAPbI_3$. (**e**) The band structure and (**f**) the DOS of δ-$FAPbI_3$. The calculations of the electronic properties were based on density functional theory with the hybrid Heyd–Scuseria–Ernzerhof functional and include spin–orbit coupling (HSE06-SOC).

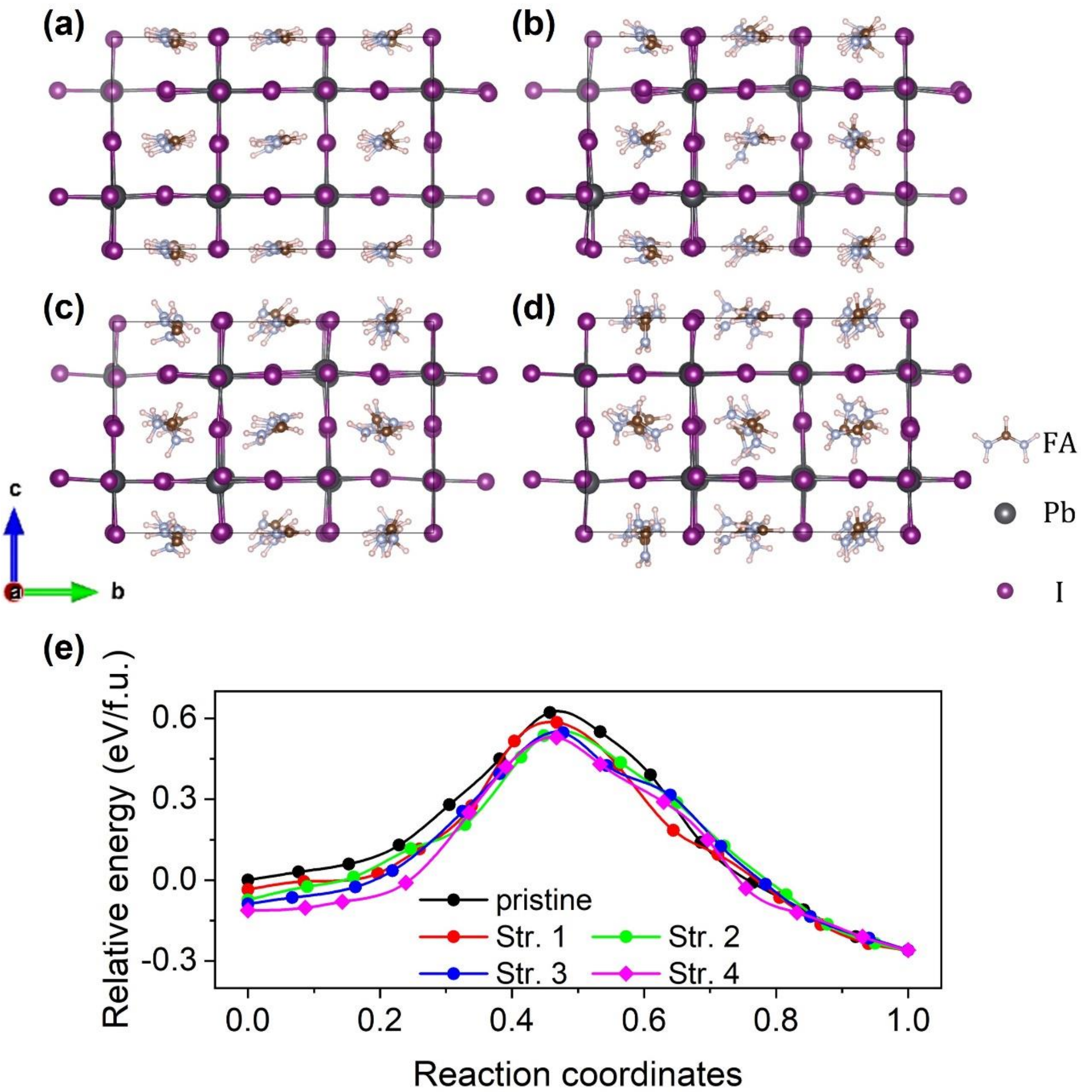

Supplementary Figure 2. α-δ phase transition of $FAPbI_3$ with FA-cationic disorder. Atomic structures of $FAPbI_3$ perovskite with FA-cationic disorder in various degrees extracted from *ab initio* molecular dynamic (AIMD) simulations at room temperature, namely, (**a**) Str.1, (**b**) Str.2, (**c**) Str.1, and (**d**) Str.4. Noted that from Str.1 to Str.4, the orientational disorder of the FA cations is increasingly more significant compared with the pristine structure in **Figure S1a**. (**e**) Evolution of and the potential energy as a function of reaction coordinate of α-δ phase transition of pristine $FAPbI_3$ and those with FA-cationic disorder (Str.1-Str.4).

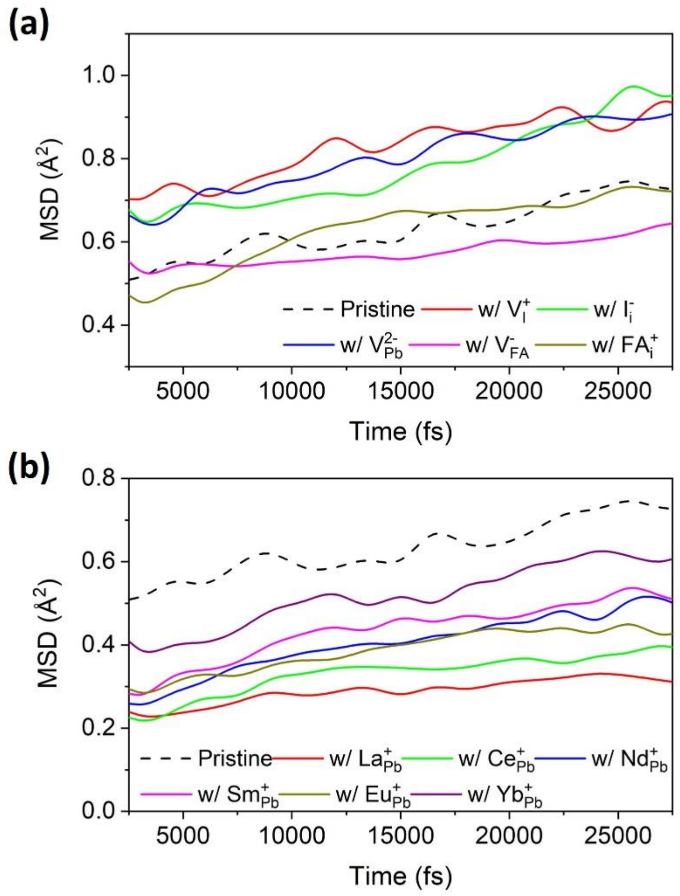

Supplementary Figure 3. MSD from AIMD simulation. Mean square displacement (MSD) of the inorganic skeleton within 30 ps during AIMD simulations at 300 K: (**a**) for pristine and systems containing intrinsic defects, and (**b**) for pristine and Ln-doped systems.

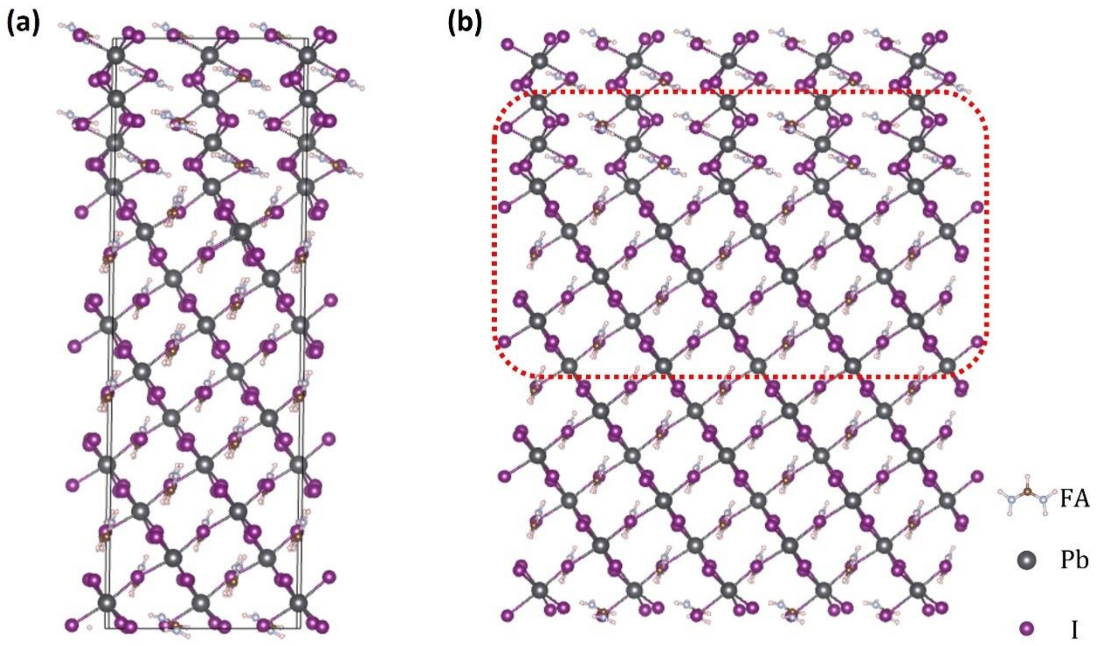

Supplementary Figure 4. Atomic configuration of α-δ interface models. The 312-atom (**a**) and 1248-atom (**b**) interface models between α-$FAPbI_3$ (111) and δ-$FAPbI_3$ (100) phases.

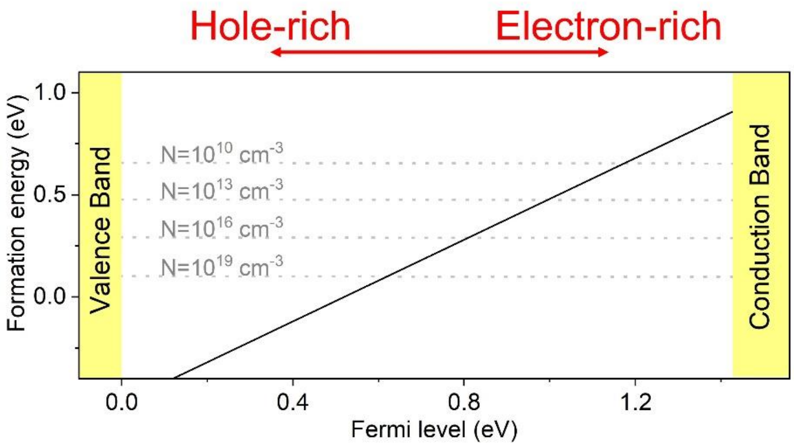

Supplementary Figure 5. Formation energy of V_I. The formation energy of V_I in $FAPbI_3$ calculated under the I-moderate growth conditions, as a function of the Fermi level. The related defect densities at room temperature are denoted.

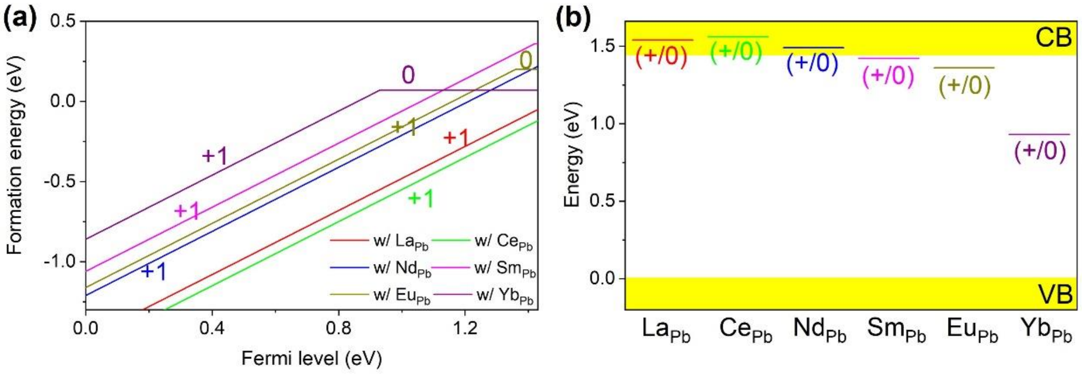

Supplementary Figure 6. Formation energies and Transition energy levels. (**a**) Formation energies of Pb substituted by lanthanide ions (namely $\mathrm{La_{Pb}}$, $\mathrm{Ce_{Pb}}$, $\mathrm{Nd_{Pb}}$, $\mathrm{Sm_{Pb}}$, $\mathrm{Eu_{Pb}}$, and $\mathrm{Yb_{Pb}}$) in $FAPbI_3$, as a function of the Fermi level. (**b**) Transition energy levels of Pb substituted by Ln ions are plotted relative to the valence and conduction band edges of $FAPbI_3$.

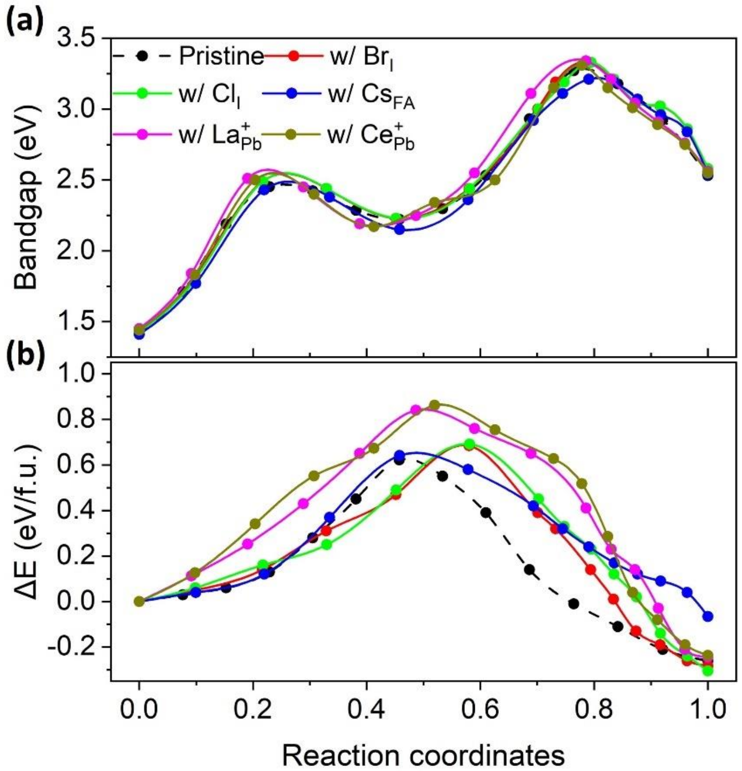

Supplementary Figure 7. α-δ phase transition in Cs-, Br-, Cl-, La-, and Ce-doped $FAPbI_3$. The evolution of the (**a**) bandgap and (**b**) potential energy as a function of the reaction coordinate of the α-δ phase transition in the representative Cs-, Br-, Cl-, La-, and Ce-doped $FAPbI_3$.

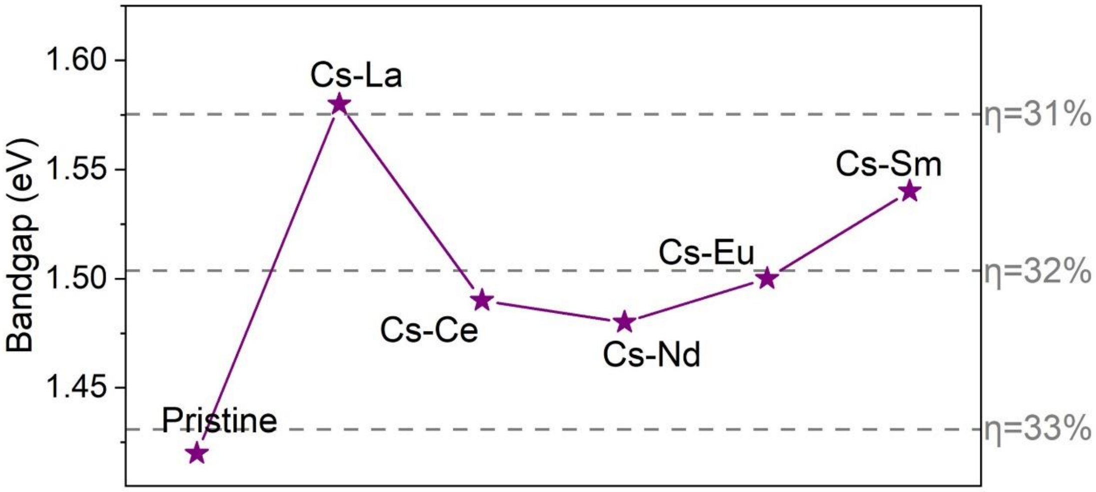

Supplementary Figure 8. Bandgaps for A-B mixed-doped $FAPbI_3$. Calculated electronic bandgaps for pristine and A-B mixed-doped (namely, Cs-La, Cs-Ce, Cs-Nd, Cs-Eu, and Cs-Sm) $FAPbI_3$ based on HSE-SOC scheme. The corresponding photovoltaic efficiency limits were presented based on the detailed balance limit theory.

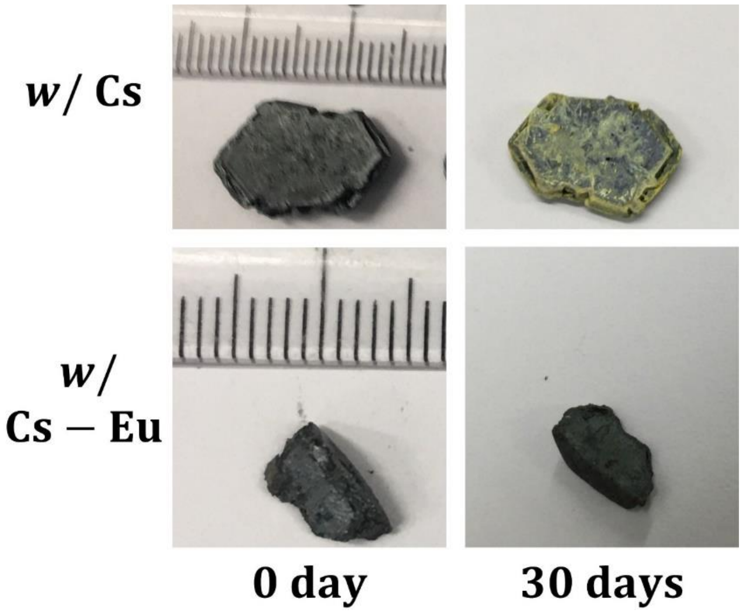

Supplementary Figure 9. Stability of Cs and Cs-Eu doped $FAPbI_3$ single-crystal samples. Photographs of the Cs-doped and Cs-Eu mixed-doped $FAPbI_3$ single-crystal samples, before and after a 30-day air exposure, respectively.

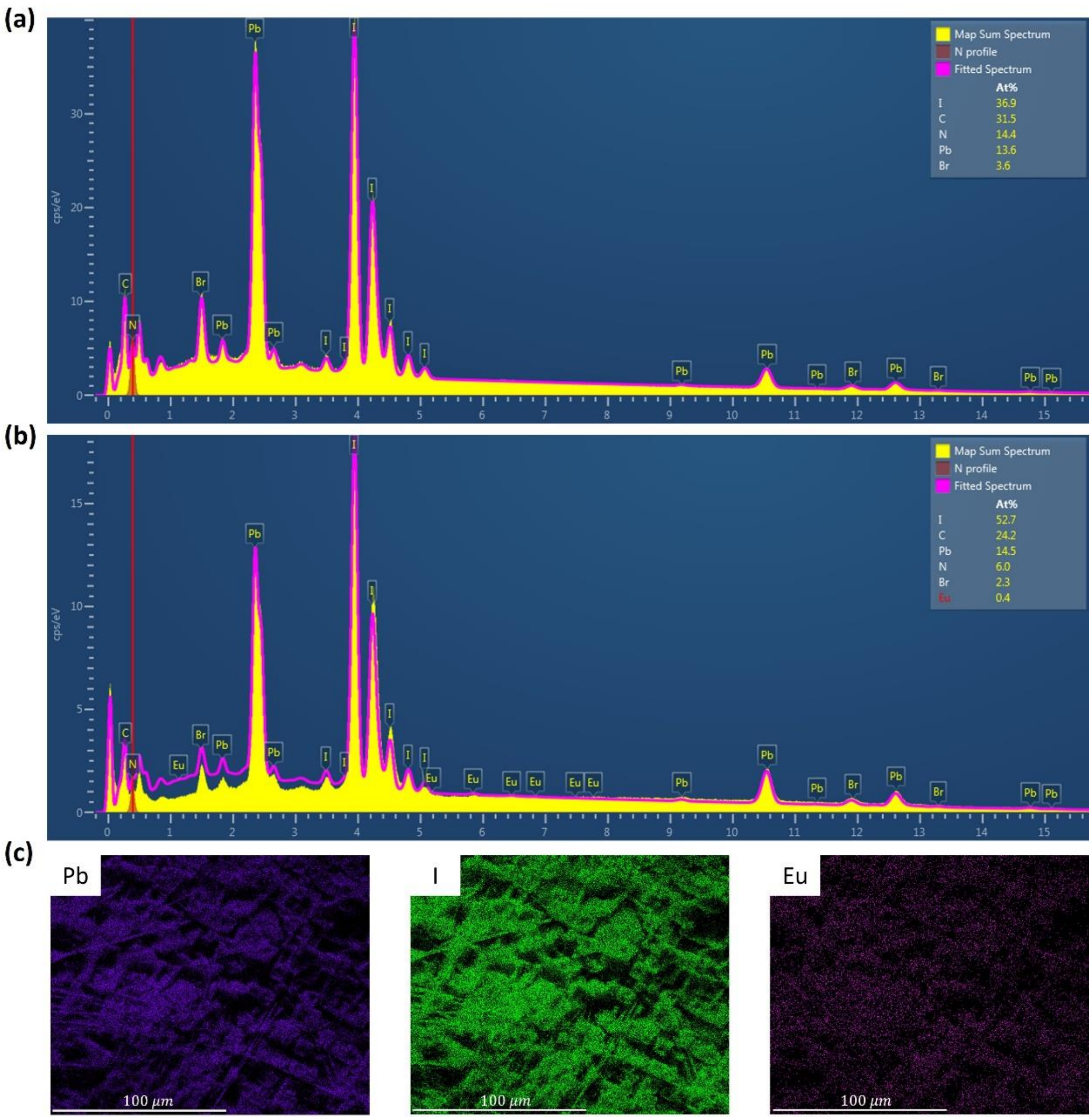

Supplementary Figure 10. EDS of Cs and Cs-Eu doped $FAPbI_3$. EDS atomic composition of (**a**) Cs-doped and (**b**) Cs-Eu mixed-doped $FAPbI_3$ perovskite single crystals. (**c**) EDS mapping of Pb, I, Eu in the Cs-Eu mixed-doped $FAPbI_3$ perovskite single crystal.

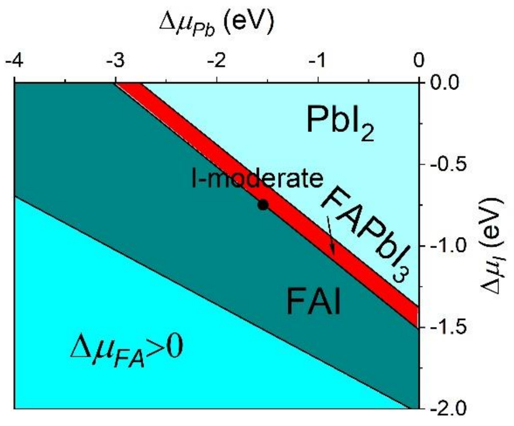

Supplementary Figure 11. Phase map of $FAPbI_3$. Phase map for thermal equilibrium growth condition of $FAPbI_3$. The relative chemical potentials $\Delta\mu_i$ of component atoms (FA, Pb, I) should satisfy:

$$\Delta\mu_{FA} + \Delta\mu_{Pb} + 3\Delta\mu_I = \Delta H_f(FAPbI_3)$$

$$\Delta\mu_{FA} + \Delta\mu_I < \Delta H_f(FAI)$$

$$\Delta\mu_{Pb} + 2\Delta\mu_I < \Delta H_f(PbI_2)$$

$$\Delta\mu_{FA} < 0;\ \Delta\mu_{Sn} < 0;\ \Delta\mu_{Br} < 0$$

where the $FAPbI_3$ will be thermodynamically stable instead of its secondary phases or element phases of components. With all these conditions, the relative chemical potentials $\Delta\mu_i$ are restricted in red area. In this study, we chose the I-moderate growth conditions for further evaluating the defect formation energies.
